# Hospitalizations of Children and Adolescents with Laboratory-Confirmed COVID-19 — COVID-NET, 14 States, July 2021–January 2022

**DOI:** 10.15585/mmwr.mm7107e4

**Published:** 2022-02-18

**Authors:** Kristin J. Marks, Michael Whitaker, Onika Anglin, Jennifer Milucky, Kadam Patel, Huong Pham, Shua J. Chai, Pam Daily Kirley, Isaac Armistead, Sarah McLafferty, James Meek, Kimberly Yousey-Hindes, Evan J. Anderson, Kyle P. Openo, Andy Weigel, Justin Henderson, Val Tellez Nunez, Kathryn Como-Sabetti, Ruth Lynfield, Susan L. Ropp, Chad Smelser, Grant R. Barney, Alison Muse, Nancy M. Bennett, Sophrena Bushey, Laurie M. Billing, Eli Shiltz, Nasreen Abdullah, Melissa Sutton, William Schaffner, H. Keipp Talbot, Ryan Chatelain, Andrea George, Christopher A. Taylor, Meredith L. McMorrow, Cria G. Perrine, Fiona P. Havers, COVID-NET Surveillance Team, COVID-NET Surveillance Team, Arthur Reingold, Nisha Alden, Breanna Kawasaki, Maria Correa, Carol Lyons, Emily Fawcett, Katelyn Ward, Kayla Bilski, Erica Bye, Emily B. Hancock, Murtada Khalifa, Adam Rowe, Nancy Spina, Virginia Cafferky, Kevin Popham, Sam Hawkins, Tiffanie Markus, Keegan McCaffrey, Andrea Price

**Affiliations:** ^1^CDC COVID-19 Emergency Response Team; ^2^Epidemic Intelligence Service, CDC; ^3^General Dynamics Information Technology, Atlanta, Georgia; ^4^California Emerging Infections Program, Oakland, California; ^5^Career Epidemiology Field Officer Program, CDC; ^6^Colorado Department of Public Health and Environment; ^7^Connecticut Emerging Infections Program, Yale School of Public Health, New Haven, Connecticut; ^8^Emory University School of Medicine, Atlanta, Georgia; ^9^Georgia Emerging Infections Program, Georgia Department of Public Health; ^10^Atlanta Veterans Affairs Medical Center, Atlanta, Georgia; ^11^Iowa Department of Public Health; ^12^Michigan Department of Health and Human Services; ^13^Minnesota Department of Health; ^14^New Mexico Department of Health; ^15^New York State Department of Health; ^16^University of Rochester School of Medicine and Dentistry, Rochester, New York; ^17^Ohio Department of Health; ^18^Public Health Division, Oregon Health Authority; ^19^Vanderbilt University Medical Center, Nashville, Tennessee; ^20^Salt Lake County Health Department, Salt Lake City, Utah.; University of California; Berkeley; Berkeley; California; Colorado Department of Public Health and Environment; Colorado Department of Public Health and Environment; Connecticut Emerging Infections Program; Yale School of Public Health; New Haven; Connecticut; Connecticut Emerging Infections Program; Yale School of Public Health; New Haven; Connecticut; Georgia Emerging Infections Program; Georgia Department of Public Health,; Atlanta; Georgia; Veterans Affairs Medical Center; Atlanta, Georgia; Foundation for Atlanta Veterans Education and Research, Decatur, Georgia; Georgia Emerging Infections Program; Georgia Department of Public Health, Atlanta; Georgia; Division of Infectious Diseases; Emory University School of Medicine; Atlanta; Georgia; Minnesota Department of Health; Minnesota Department of Health; University of New Mexico Health Sciences Center; New Mexico Emerging Infections Program; Albuquerque; New Mexico; CDC Foundation; Atlanta; Georgia; New Mexico Department of Health; New York State Department of Health; New York State Department of Health; University of Rochester School of Medicine and Dentistry; Rochester; New York; University of Rochester School of Medicine and Dentistry; Rochester; New York; Public Health Division; Oregon Health Authority; Vanderbilt University Medical Center; Nashville; Tennessee; Utah Department of Health; Salt Lake County Health Department; Salt Lake City; Utah

The first U.S. case of COVID-19 attributed to the Omicron variant of SARS-CoV-2 (the virus that causes COVID-19) was reported on December 1, 2021 (*1*), and by the week ending December 25, 2021, Omicron was the predominant circulating variant in the United States.* Although COVID-19–associated hospitalizations are more frequent among adults,^†^ COVID-19 can lead to severe outcomes in children and adolescents (*2*). This report analyzes data from the Coronavirus Disease 19–Associated Hospitalization Surveillance Network (COVID-NET)^§^ to describe COVID-19–associated hospitalizations among U.S. children (aged 0–11 years) and adolescents (aged 12–17 years) during periods of Delta (July 1–December 18, 2021) and Omicron (December 19, 2021–January 22, 2022) predominance. During the Delta- and Omicron-predominant periods, rates of weekly COVID-19–associated hospitalizations per 100,000 children and adolescents peaked during the weeks ending September 11, 2021, and January 8, 2022, respectively. The Omicron variant peak (7.1 per 100,000) was four times that of the Delta variant peak (1.8), with the largest increase observed among children aged 0–4 years.^¶^ During December 2021, the monthly hospitalization rate among unvaccinated adolescents aged 12–17 years (23.5) was six times that among fully vaccinated adolescents (3.8). Strategies to prevent COVID-19 among children and adolescents, including vaccination of eligible persons, are critical.**

COVID-NET conducts population-based surveillance for laboratory-confirmed COVID-19–associated hospitalizations in 99 counties across 14 states.^††^ Among residents of a predefined surveillance catchment area, COVID-19–associated hospitalizations are defined as receipt of a positive SARS-CoV-2 real-time reverse transcription–polymerase chain reaction (RT-PCR) or rapid antigen detection test result during hospitalization or during the 14 days before admission. This analysis describes weekly hospitalization rates during the weeks ending July 3, 2021–January 22, 2022, to coincide with a period during which detailed clinical data (e.g., intensive care unit [ICU] admission) were available (monthly, July 1–December 31, 2021). Unadjusted weekly COVID-19–associated hospitalization rates were calculated by dividing the total number of hospitalized patients by the population estimates within each age group for the counties included in the surveillance catchment area.^§§^ ICU admission rates were similarly calculated using 2-week periods. All rates were estimated per 100,000 population for children, adolescents, or both.

Among adolescents aged 12–17 years*,* hospitalization rates were calculated by COVID-19 vaccination status, which was determined both for hospitalized patients and the catchment population using linkage to state immunization information systems data.^¶¶^ Monthly incidence was calculated by summing the total number of hospitalized adolescents who were fully vaccinated (≥14 days after final dose in primary series) for each day of the month and dividing by the sum of fully vaccinated adolescents in the underlying population for each day of the month; the same method was used to calculate incidence in unvaccinated adolescents.*** Rate ratios (RRs) and 95% CIs were calculated.

Trained surveillance staff members conducted medical chart abstractions for all pediatric COVID-NET patients using a standardized case report form through November 2021. Because of the large number of cases during December 2021, some sites examined clinical outcome data on a representative sample of hospitalized children.^†††^ Data on indicators of severe disease were collected (i.e., hospital length of stay, ICU admission, use of invasive mechanical ventilation [IMV],^§§§^ and in-hospital death), as were data on primary reason for admission^¶¶¶^ and symptoms that were present when the patient was admitted**** (*3*). Proportions were compared between periods of Delta predominance (July 1–December 18, 2021) and Omicron predominance (December 19–31, 2021); a variant that accounted for >50% of sequenced isolates was considered to be predominant.^††††^ A similar analysis was completed by vaccination status among adolescents, the only pediatric age group for whom a COVID-19 vaccine had been approved throughout the surveillance period. Wilcoxon rank-sum tests were used to compare medians, and chi-square or Fisher’s exact tests were used to compare proportions; p-values <0.05 were considered statistically significant. Percentages were weighted to account for the probability of selection for sampled cases and further adjusted to account for nonresponse (defined as an incomplete chart review). Data were analyzed using SAS (version 9.4; SAS Institute). This activity was reviewed by CDC and was conducted consistent with applicable federal law and CDC policy.^§§§§^

During the Delta- and Omicron-predominant periods, pediatric weekly hospitalization rates peaked during the weeks ending September 11, 2021, and January 8, 2022, respectively; the Omicron variant peak (7.1 per 100,000 children and adolescents) was four times that of the Delta variant peak (1.8). Hospitalization rates among children aged 0–4 years were approximately five times as high during the peak week of the Omicron period (15.6) than during the Delta period (2.9) (RR = 5.4; 95% CI = 4.0–7.2) (Figure); RRs were also increased among children aged 5–11 years (Delta = 1.1; Omicron = 2.4; RR = 2.3; 95% CI = 1.5–3.6) and adolescents aged 12–17 years (Delta = 1.7; Omicron = 5.9; RR = 3.5; 95% CI = 2.5–5.0). Peak ICU admission rates for children and adolescents were 1.4 times higher during Omicron predominance (2-week period ending December 31, 2021 [1.5]) than during Delta predominance (2-week period ending September 11, 2021 [1.1]). During December 2021, when both variants were circulating, the rates of hospitalization were 23.5 and 3.8 per 100,000 among unvaccinated and fully vaccinated adolescents, respectively (RR = 6.3; 95% CI = 4.4–8.6).

**FIGURE:**
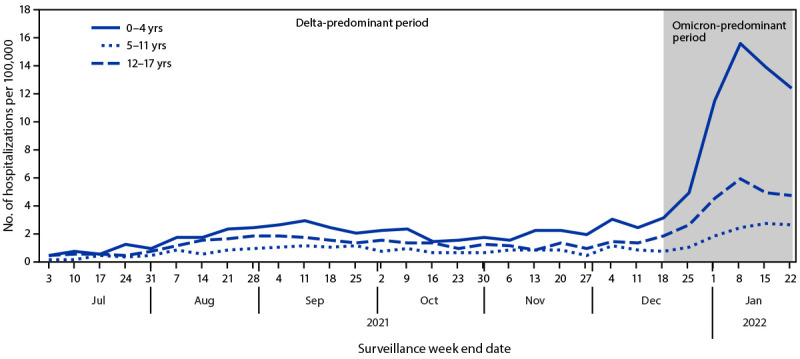
Weekly COVID-19–associated hospitalization rates* among children and adolescents aged 0–17 years, by age group — COVID-NET, 14 states,^†^ July 3, 2021–January 22, 2022 **Abbreviation:** COVID-NET = Coronavirus Disease 2019–Associated Hospitalization Surveillance Network. * Number of patients with laboratory-confirmed COVID-19–associated hospitalizations per 100,000 population; rates are subject to change as additional data are reported. ^†^ COVID-NET sites are in the following 14 states: California, Colorado, Connecticut, Georgia, Iowa, Maryland, Michigan, Minnesota, New Mexico, New York, Ohio, Oregon, Tennessee, and Utah. Starting the week ending December 4, 2021, Maryland data are removed from weekly rate calculations.

Complete clinical data were available for 1,834^¶¶¶¶^ and 266***** hospitalized children and adolescents in the Delta-predominant (July 1–December 18, 2021) and Omicron-predominant (December 19, 2021–December 31, 2021) periods, respectively. The proportions of hospitalized children and adolescents requiring ICU admission (Delta = 27.8%; Omicron = 20.2%) or IMV (Delta = 6.3%; Omicron = 2.3%) were significantly lower during the Omicron period (Table 1). No significant difference was detected between the Delta- and Omicron-predominant periods in the proportion of patients with COVID-19–related symptoms recorded at admission (87.7% versus 86.9%) or with COVID-19 as the primary reason for admission (81.3% versus 81.6%).

**TABLE 1:** Demographic and clinical characteristics and outcomes among children and adolescents aged 0–17 years with laboratory-confirmed COVID-19–associated hospitalizations,* by date of admission — COVID-NET, 14 states,^†^ July 1–December 31, 2021

Characteristic	No. of hospitalized children (%)			p-value^§^
	Total	Jul 1–Dec 18	Dec 19–31	
	Jul 1–Dec 31			
**Total**	**2,100 (100.0)^¶^**	**1,834 (82.3)^¶^**	**266 (17.7)^¶^**	**—**
**Age, yrs, median (IQR)**	**7 (1–14)**	7 (1–14)	3.5 (0.4–13)	<0.001
**Age group, yrs**				
0–4	**920 (44.6)**	778 (42.5)	142 (54.2)	0.003
5–11	**460 (21.5)**	417 (22.5)	43 (16.9)	
12–17	**720 (33.9)**	639 (34.9)	81 (28.9)	
**Sex**				
Male	**1,081 (51.7)**	934 (51.2)	147 (54.2)	0.38
Female	**1,019 (48.3)**	900 (48.8)	119 (45.8)	
Race and ethnicity**				
Hispanic	**463 (21.8)**	420 (23.1)	43 (15.7)	<0.001
Black, non-Hispanic	**736 (35.8)**	619 (33.4)	117 (47.1)	
White, non-Hispanic	**670 (31.3)**	598 (32.6)	72 (25.5)	
Asian or Pacific Islander, non-Hispanic	**82 (3.9)**	71 (3.9)	11 (3.7)	
All other races^††^	**47 (2.3)**	41 (2.3)	6 (2.1)	
Unknown race and ethnicity	**102 (5.0)**	85 (4.8)	17 (5.9)	
**Primary reason for admission^§§^**				
Likely related to COVID-19	**1,703 (81.3)**	1,489 (81.3)	214 (81.6)	0.19
Obstetrics	**63 (2.9)**	57 (3.0)	6 (2.2)	
Inpatient surgery	**53 (2.6)**	43 (2.5)	10 (3.3)	
Psychiatric admission requiring medical care	**118 (5.6)**	108 (5.9)	10 (4.0)	
Trauma	**75 (3.5)**	67 (3.7)	8 (2.8)	
Other reason	**78 (3.8)**	62 (3.3)	16 (6.1)	
Unknown reason	**6 (0.3)**	6 (0.3)	0 (—)	
**COVID-19–related symptoms at admission^¶¶^**				
Yes	**1,832 (87.6)**	1,604 (87.7)	228 (86.9)	0.72
No	**264 (12.4)**	228 (12.3)	36 (13.1)	
**Hospitalization outcomes**				
Length of hospital stay, days, median (IQR)	**3 (1–5)**	3 (2–5)	2 (1–5)	0.15
ICU admission***	**562 (26.4)**	510 (27.8)	52 (20.2)	0.01
Invasive mechanical ventilation***	**118 (5.6)**	112 (6.3)	6 (2.3)	0.01
In-hospital death	**11 (0.5)**	11 (0.6)	0 (—)	0.38
**Vaccination status (among patients aged 12–17 yrs)**				
Fully vaccinated^†††^	**71 (9.9)**	53 (8.3)	18 (22.2)	<0.001
Unvaccinated	**647 (90.1)**	584 (91.7)	63 (77.8)	

**Abbreviations:** COVID-NET = Coronavirus Disease 2019–Associated Hospitalization Surveillance Network; ICU = intensive care unit.

* Data are from a weighted sample of hospitalized children and adolescents with completed medical record abstractions. Sample sizes presented are unweighted with weighted percentages. ^†^ Includes persons admitted to a hospital with an admission date during July 1–December 31, 2021. Maryland contributed data through November 26, 2021. Counties included in COVID-NET surveillance: California (Alameda, Contra Costa, and San Francisco counties); Colorado (Adams, Arapahoe, Denver, Douglas, and Jefferson counties); Connecticut (Middlesex and New Haven counties); Georgia (Clayton, Cobb, DeKalb, Douglas, Fulton, Gwinnett, Newton, and Rockdale counties); Iowa (one county represented); Maryland (Allegany, Anne Arundel, Baltimore, Baltimore City, Calvert, Caroline, Carroll, Cecil, Charles, Dorchester, Frederick, Garrett, Harford, Howard, Kent, Montgomery, Prince George’s, Queen Anne’s, St. Mary’s, Somerset, Talbot, Washington, Wicomico, and Worcester counties); Michigan (Clinton, Eaton, Genesee, Ingham, and Washtenaw counties); Minnesota (Anoka, Carver, Dakota, Hennepin, Ramsey, Scott, and Washington counties); New Mexico (Bernalillo, Chaves, Doña Ana, Grant, Luna, San Juan, and Santa Fe counties); New York (Albany, Columbia, Genesee, Greene, Livingston, Monroe, Montgomery, Ontario, Orleans, Rensselaer, Saratoga, Schenectady, Schoharie, Wayne, and Yates counties); Ohio (Delaware, Fairfield, Franklin, Hocking, Licking, Madison, Morrow, Perry, Pickaway, and Union counties); Oregon (Clackamas, Multnomah, and Washington counties); Tennessee (Cheatham, Davidson, Dickson, Robertson, Rutherford, Sumner, Williamson, and Wilson counties); and Utah (Salt Lake County).

^§^ Proportions between the Delta and Omicron predominance periods were compared with chi-square tests or Fisher’s exact tests (as appropriate), and medians were compared with the Wilcoxon rank-sum test; p-values <0.05 were considered statistically significant.

^¶^ Data are missing for <5% of observations for all variables.

** If ethnicity was unknown, non-Hispanic ethnicity was assumed.

^††^ Includes non-Hispanic persons reported as other or multiple races.

^§§^ Among sampled patients, COVID-NET collects data on the primary reason for admission to differentiate hospitalizations of patients with laboratory-confirmed SARS-CoV-2 infection who are likely admitted primarily for COVID-19 illness rather than for other reasons. During chart review, if the surveillance officer finds that the chief complaint or history of present illness mentions fever or respiratory illness, COVID-19–like illness, or suspected COVID-19, then the case is categorized as COVID-19–related illness as the primary reason for admission. Reasons for admission that are likely primarily not related to COVID-19 include the following categories: obstetrics/labor and delivery, inpatient surgery or procedures, psychiatric admission requiring acute medical care, trauma, other, or unknown. Reasons categorized as “other” are reviewed by two physicians to determine whether the admission is likely COVID-19 related.

^¶¶^ COVID-19–related symptoms included respiratory symptoms (congestion or runny nose, cough, hemoptysis or bloody sputum, shortness of breath or respiratory distress, sore throat, upper respiratory infection, influenza-like illness, and wheezing) and nonrespiratory symptoms (abdominal pain, altered mental status or confusion, anosmia or decreased smell, chest pain, conjunctivitis, diarrhea, dysgeusia or decreased taste, fatigue, fever or chills, headache, muscle aches or myalgias, nausea or vomiting, rash, and seizures), and among those aged <2 years, included apnea, cyanosis, decreased vocalization or stridor, dehydration, hypothermia, inability to eat or poor feeding, and lethargy. Symptoms are abstracted from the medical chart and might not be complete.

*** ICU admission and invasive mechanical ventilation are not mutually exclusive categories, and patients could have received both.

^†††^ Fully vaccinated adolescents with COVID-19–associated hospitalizations were defined as those who had received the final dose in their primary series ≥14 days before receiving a positive SARS-CoV-2 test result associated with their hospitalization. Adolescents who received only 1 vaccine dose ≥14 days before the SARS-CoV-2 test date or had received a single dose of vaccine <14 days before the positive SARS-CoV-2 test results were considered partially vaccinated; they were not included in rates and were grouped with unvaccinated adolescents in other analyses. COVID-NET sites, through agreements with state health departments and other partners, collect COVID-19 vaccination information on COVID-19–associated hospitalizations through state-based vaccine registries. When possible, sites collect COVID-19 vaccination status on all persons with COVID-19 cases who are hospitalized, including the number of vaccine doses received, the vaccine product, and dates of vaccination administration.

The proportion of hospitalized adolescents who were fully vaccinated was significantly lower during the Delta-predominant period (8.3%) than during the Omicron-predominant period (22.2%) (Table 1), consistent with increasing adolescent vaccination coverage during the surveillance period. During July 1–December 31, 2021, 42.4% of hospitalized unvaccinated adolescents were non-Hispanic Black adolescents (Table 2). A higher proportion of unvaccinated adolescents (70.3%) than fully vaccinated adolescents (40.8%) had COVID-19 as a primary reason for admission. A significantly higher proportion of unvaccinated adolescents were admitted to the ICU (30.3%) than were those who were vaccinated (15.5%).

**TABLE 2:** Demographic and clinical characteristics and outcomes among fully vaccinated* and unvaccinated adolescents aged 12–17 years with laboratory-confirmed COVID-19–associated hospitalizations,^†^ by date of admission — COVID-NET, 14 states,^§^ July 1–December 31, 2021

Characteristic	No. of hospitalized adolescents (%)						
	Unvaccinated	Vaccinated	p-value^¶^	Unvaccinated		Vaccinated	
	Total						
	Jul 1–Dec 31			Jul 1–Dec 18	Dec 19–31	Jul 1–Dec 18	Dec 19–31
**Total**	647 (100.0)**	71 (100.0)**	**—**	584 (90.2)**	63 (9.8)**	53 (74.6)**	18 (25.4)**
**Age, yrs, median (IQR)**	**15 (14–16)**	**15 (14–16)**	**0.75**	**15 (14–17)**	**14 (13–16)**	**15 (14–16)**	**15 (14–16)**
**Sex**							
Male	**298 (46.0)**	**25 (35.2)**	0.08	266 (45.5)	32 (50.8)	20 (37.7)	5 (27.7)
Female	**349 (54.0)**	**46 (64.8)**		318 (54.5)	31 (49.2)	33 (62.3)	13 (72.3)
**Race and ethnicity^††^**							
Hispanic	**148 (22.9)**	**14 (19.7)**	0.006	136 (23.3)	12 (19.0)	12 (22.6)	2 (11.1)
Black, non-Hispanic	**274 (42.4)**	**17 (24.0)**		240 (41.1)	34 (53.9)	11 (20.8)	6 (33.4)
White, non-Hispanic	**162 (25.0)**	**32 (45.0)**		154 (26.4)	8 (12.7)	25 (47.1)	7 (38.8)
Asian or Pacific Islander, non-Hispanic	**14 (2.2)**	**1 (1.4)**		10 (1.7)	4 (6.4)	1 (1.9)	0 (—)
All other races^§§^	**20 (3.1)**	**2 (2.8)**		19 (3.3)	1 (1.6)	1 (1.9)	1 (5.5)
Unknown race and ethnicity	**29 (4.5)**	**5 (7.1)**		25 (4.3)	4 (6.4)	3 (5.7)	2 (11.2)
**Primary reason for admission^¶¶^**							
Likely related to COVID-19	**454 (70.3)**	**29 (40.8)**	<0.001	413 (70.8)	41 (65.0)	19 (35.8)	10 (55.5)
Obstetrics	**40 (6.2)**	**0 (—)**		36 (6.2)	4 (6.4)	0 (—)	0 (—)
Inpatient surgery	**15 (2.3)**	**7 (9.8)**		13 (2.2)	2 (3.2)	5 (9.4)	2 (11.1)
Psychiatric admission requiring medical care	**79 (12.2)**	**27 (38.1)**		72 (12.4)	7 (11.1)	24 (45.3)	3 (16.8)
Trauma	**40 (6.2)**	**4 (5.6)**		35 (6.0)	5 (8.0)	3 (5.7)	1 (5.5)
Other reason	**16 (2.5)**	**4 (5.6)**		12 (2.1)	4 (6.3)	2 (3.8)	2 (11.1)
Unknown reason	**2 (0.3)**	**0 (—)**		2 (0.3)	0 (—)	0 (—)	0 (—)
COVID-19–related symptoms at admission***							
Yes	**536 (83.0)**	**53 (74.6)**	0.08	487 (83.4)	49 (79.0)	37 (69.8)	16 (88.9)
No	**110 (17.0)**	**18 (25.4)**		97 (16.6)	13 (21.0)	16 (30.2)	2 (11.1)
**Hospitalization outcomes**							
Length of hospital stay, days, median (IQR)	**4 (2–7)**	**3 (1–8)**	0.55	4 (2–6.5)	4 (2–8)	3 (2–9)	3 (1–5)
ICU admission^†††^	**196 (30.3)**	**11 (15.5)**	0.009	184 (31.6)	12 (19.1)	8 (15.1)	3 (16.6)
Invasive mechanical ventilation^†††^	**42 (6.5)**	**6 (8.4)**	0.54	41 (7.1)	1 (1.6)	5 (9.4)	1 (5.5)
In-hospital death	**5 (0.8)**	**2 (2.8)**	0.10	5 (0.9)	0 (—)	2 (3.8)	0 (—)

**Abbreviations:** COVID-NET = Coronavirus Disease 2019–Associated Hospitalization Surveillance Network; ICU = intensive care unit.

* Fully vaccinated adolescents with COVID-19–associated hospitalizations were defined as those who had received the final dose in their primary series ≥14 days before receiving a positive SARS-CoV-2 test result associated with their hospitalization. Adolescents who received only 1 vaccine dose ≥14 days before the SARS-CoV-2 test date or had received a single dose of vaccine <14 days before the positive SARS-CoV-2 test results were considered partially vaccinated; they were not included in rates and were grouped with unvaccinated adolescents in other analyses. COVID-NET sites, through agreements with state health departments and other partners, collect COVID-19 vaccination information on COVID-19–associated hospitalizations through state-based vaccine registries. When possible, sites collect COVID-19 vaccination status on all persons with COVID-19 cases who are hospitalized, including the number of vaccine doses received, the vaccine product, and dates of vaccination administration.

^†^ Data are from a weighted sample of hospitalized children and adolescents with completed medical record abstractions. Sample sizes presented are unweighted with weighted percentages.

^§^ Includes persons admitted to a hospital with an admission date during July 1–December 31, 2021. Maryland contributed data through November 26, 2021. Counties included in COVID-NET surveillance: California (Alameda, Contra Costa, and San Francisco counties); Colorado (Adams, Arapahoe, Denver, Douglas, and Jefferson counties); Connecticut (Middlesex and New Haven counties); Georgia (Clayton, Cobb, DeKalb, Douglas, Fulton, Gwinnett, Newton, and Rockdale counties); Iowa (one county represented); Maryland (Allegany, Anne Arundel, Baltimore, Baltimore City, Calvert, Caroline, Carroll, Cecil, Charles, Dorchester, Frederick, Garrett, Harford, Howard, Kent, Montgomery, Prince George’s, Queen Anne’s, St. Mary’s, Somerset, Talbot, Washington, Wicomico, and Worcester counties); Michigan (Clinton, Eaton, Genesee, Ingham, and Washtenaw counties); Minnesota (Anoka, Carver, Dakota, Hennepin, Ramsey, Scott, and Washington counties); New Mexico (Bernalillo, Chaves, Doña Ana, Grant, Luna, San Juan, and Santa Fe counties); New York (Albany, Columbia, Genesee, Greene, Livingston, Monroe, Montgomery, Ontario, Orleans, Rensselaer, Saratoga, Schenectady, Schoharie, Wayne, and Yates counties); Ohio (Delaware, Fairfield, Franklin, Hocking, Licking, Madison, Morrow, Perry, Pickaway, and Union counties); Oregon (Clackamas, Multnomah, and Washington counties); Tennessee (Cheatham, Davidson, Dickson, Robertson, Rutherford, Sumner, Williamson, and Wilson counties); and Utah (Salt Lake County).

^¶^ Proportions between vaccinated and unvaccinated adolescents were compared with chi-square tests or Fisher’s exact tests (as appropriate), and medians were compared with the Wilcoxon rank sum test; p-values <0.05 were considered statistically significant.

** Data are missing for <5% of observations for all variables.

^††^ If ethnicity was unknown, non-Hispanic ethnicity was assumed.

^§§^ Includes non-Hispanic persons reported as other or multiple races.

^¶¶^ Among sampled patients, COVID-NET collects data on the primary reason for admission to differentiate hospitalizations of patients with laboratory-confirmed SARS-CoV-2 infection who are likely admitted primarily for COVID-19 illness rather than for other reasons. During chart review, if the surveillance officer finds that the chief complaint or history of present illness mentions fever or respiratory illness, COVID-19–like illness, or suspected COVID-19, then the case is categorized as COVID-19–related illness as the primary reason for admission. Reasons for admission that are likely primarily not related to COVID-19 include the following categories: obstetrics/labor and delivery, inpatient surgery or procedures, psychiatric admission requiring acute medical care, trauma, other, or unknown. Reasons categorized as “other” are reviewed by two physicians to determine whether the admission is likely COVID-19 related.

*** COVID-19–related symptoms included respiratory symptoms (congestion or runny nose, cough, hemoptysis or bloody sputum, shortness of breath or respiratory distress, sore throat, upper respiratory infection, influenza-like illness, and wheezing) and nonrespiratory symptoms (abdominal pain, altered mental status or confusion, anosmia or decreased smell, chest pain, conjunctivitis, diarrhea, dysgeusia or decreased taste, fatigue, fever or chills, headache, muscle aches or myalgias, nausea or vomiting, rash, and seizures), and among those aged <2 years, included apnea, cyanosis, decreased vocalization or stridor, dehydration, hypothermia, inability to eat or poor feeding, and lethargy. Symptoms are abstracted from the medical chart and might not be complete.

^†††^ ICU admission and invasive mechanical ventilation are not mutually exclusive categories, and patients could have received both.

## Discussion

The Omicron variant, which is associated with increased transmissibility and partial escape from infection- or vaccine-induced immunity, replaced Delta as the predominant variant in the United States in late December 2021 (*1*). Once the Omicron variant became predominant, peak population-based COVID-19–associated hospitalization rates among children and adolescents were four times as high as rates during the peak of the Delta period. Children aged 0–4 years, who were ineligible for vaccination during this time, experienced the largest increase in hospitalization rates. Observed indicators of severe COVID-19 among children and adolescents, in addition to the potential for longer-term sequelae (*4*,*5*), highlight the importance of multicomponent strategies to reduce the incidence of COVID-19, including vaccination of eligible persons and other prevention measures.^†††††^

Among adolescents aged 12–17 years, the only pediatric age group for whom a COVID-19 vaccine was approved throughout the study period, December hospitalization rates among unvaccinated adolescents were approximately six times those among fully vaccinated adolescents, suggesting that vaccines were highly effective in preventing serious COVID-19 illness. Vaccination eligibility was expanded to include children aged 5–11 years on November 2, 2021. As of December 31, 2021, 54% of the population aged 12–17 years and 16% of those aged 5–11 years had completed a COVID-19 primary vaccination series.^§§§§§^ Increasing vaccination coverage among both age groups can reduce COVID-19–associated hospitalizations (*6*); enhanced outreach strategies are needed to address disparities in vaccination coverage by race/ethnicity.

Consistent with national hospital surveillance data (*7*), the findings in this report indicate that the Omicron-predominant period had higher rates of pediatric COVID-19 hospitalizations than the Delta-predominant period. No differences were found between the Delta- and Omicron-predominant periods in the proportion of hospitalizations that were likely to be related to COVID-19. Findings suggest that incidental admissions do not account for the increase in hospitalization rates observed during the Omicron period. Throughout the COVID-19 pandemic, admissions for reasons other than COVID-19 have been recorded (*8*,*9*), and during both the Delta- and Omicron-predominant periods, incidental admissions were more likely among fully vaccinated adolescents. Reasons for admission should continue to be monitored as more children and adolescents become fully vaccinated.

The findings in this report are subject to at least six limitations. First, COVID-19–associated hospitalizations might have been missed because of testing practices and test availability. Second, the period of Omicron variant predominance with available detailed clinical data is brief (December 19–31, 2021) and does not capture the peak of hospitalizations during the Omicron period; in addition, the Delta variant was still circulating in late December. Third, accounting for seasonality in comparisons of Delta and Omicron predominant periods was not possible. Fourth, the number of hospitalized children eligible for vaccination remained low at the time of reporting, and hospitalization rates stratified by vaccination status are subject to error if misclassification of vaccination status occurred. Fifth, because children aged 5–11 years could not meet the definition for being fully vaccinated until December 7, 2021, vaccination among this age group was not considered in this study. However, vaccinations could have affected hospitalization rates during the Omicron period. Further, boosters among adolescents aged 12–17 years could not be examined because the recommendation was so recent. Finally, the COVID-NET catchment areas include approximately 10% of the U.S. population; thus, these findings might not be generalizable to the entire United States.

Coinciding with emerging predominance of the Omicron variant, rates of COVID-19–associated hospitalization among children and adolescents increased rapidly during the last 2 weeks of December 2021, especially among those aged 0–4 years. Moreover, among adolescents, hospitalization rates were higher among those who were unvaccinated. Vaccination of eligible persons, in addition to other prevention strategies such as masking, are critical to reducing the incidence of severe COVID-19 among children and adolescents.^¶¶¶¶¶^ All persons who are eligible for vaccination should receive and stay up to date with COVID-19 vaccines to reduce the risk for severe disease for themselves and others with whom they come into contact, including children who are currently too young to be vaccinated.******

**Summary**

**What is already known about this topic?**

COVID-19 can cause severe illness in children and adolescents.

**What is added by this report?**

Coinciding with increased circulation of the Omicron variant, COVID-19–associated hospitalization rates among children and adolescents aged 0–17 years increased rapidly in late December 2021, especially among children aged 0–4 years who are not yet eligible for vaccination. Throughout the periods of Delta and Omicron predominance, hospitalization rates remained lower among fully vaccinated adolescents aged 12–17 years than among unvaccinated adolescents.

**What are the implications for public health practice?**

Strategies to prevent COVID-19 among children and adolescents, including vaccination of eligible persons, are critical.

## Footnotes

*
https://covid.cdc.gov/covid-data-tracker/#variant-proportions

† https://www.cdc.gov/coronavirus/2019-ncov/covid-data/investigations-discovery/hospitalization-death-by-age.html

§
https://www.cdc.gov/coronavirus/2019-ncov/covid-data/covid-net/purpose-methods.html

¶ COVID-NET hospitalization data are preliminary and subject to change as more data become available. In particular, case counts and rates for recent hospital admissions are subject to lag.

**
https://www.cdc.gov/coronavirus/2019-ncov/vaccines/your-vaccination.html

†† California, Colorado, Connecticut, Georgia, Iowa, Maryland, Michigan, Minnesota, New Mexico, New York, Ohio, Oregon, Tennessee, and Utah.

§§ Rates are calculated using the National Center for Health Statistics vintage 2020 bridged-race postcensal population estimates for the counties included in surveillance (https://www.cdc.gov/nchs/nvss/bridged_race.htm).

¶¶ The Food and Drug Administration granted emergency use authorization for the Pfizer-BioNTech COVID-19 vaccine for adolescents aged 12–15 years on May 10, 2021. The earliest date that adolescents in this age group could have met the definition for being a fully vaccinated COVID-19 patient was June 14, 2021.

*** Fully vaccinated adolescents with COVID-19–associated hospitalizations were defined as those who had received the final dose in their primary series ≥14 days before receiving a positive SARS-CoV-2 test result associated with their hospitalization. Adolescents who received only 1 vaccine dose ≥14 days before the SARS-CoV-2 test date or had received a single dose of vaccine <14 days before the positive SARS-CoV-2 test results were considered partially vaccinated; they were not included in rates and were grouped with unvaccinated adolescents in other analyses. Unvaccinated adolescents were defined as those who did not meet the criteria for being fully or partially vaccinated. Additional COVID-NET methods for determining vaccination status have been described previously (https://www.medrxiv.org/cgi/content/short/2021.08.27.21262356v1).

††† Colorado, Georgia, New Mexico, and Utah sampled 50% of patients during the month of December. All other sites included 100% of cases. To produce random samples of hospitalized patients for medical record abstraction, random numbers (1–100) are automatically generated and assigned to each patient as their data are entered into the surveillance database. Percentages are weighted to account for the probability of selection for sampled patients.

§§§ ICU admission and IMV are not mutually exclusive categories, and patients could have received both.

¶¶¶ Among sampled patients, COVID-NET collects data on the primary reason for admission to differentiate hospitalizations of patients with laboratory-confirmed SARS-CoV-2 infection who are likely admitted primarily for COVID-19 illness rather than for other reasons. During chart review, if the surveillance officer finds that the chief complaint or history of present illness mentions fever/respiratory illness, COVID-19-like illness, or a suspicion for COVID-19, then the case is categorized as COVID-19 related illness as the primary reason for admission.

**** COVID-19–related symptoms included respiratory symptoms (congestion or runny nose, cough, hemoptysis or bloody sputum, shortness of breath or respiratory distress, sore throat, upper respiratory infection, influenza-like illness, and wheezing) and nonrespiratory symptoms (abdominal pain, altered mental status or confusion, anosmia or decreased smell, chest pain, conjunctivitis, diarrhea, dysgeusia or decreased taste, fatigue, fever or chills, headache, muscle aches or myalgias, nausea or vomiting, rash, and seizures), and among those aged <2 years, included apnea, cyanosis, decreased vocalization or stridor, dehydration, hypothermia, inability to eat or poor feeding, and lethargy. Symptoms are abstracted from the medical chart and might not be complete.

†††† Delta became the predominant (>50%) variant circulating in the United States the week ending July 3, 2021. By the week ending December 18, 2021, Omicron accounted for 38% of circulating variants; Omicron became the predominant variant the week ending December 25 at 74%.

§§§§ 45 C.F.R. part 46.102(l)(2), 21 C.F.R. part 56; 42 U.S.C. Sect. 241(d); 5 U.S.C. Sect. 552a; 44 U.S.C. Sect. 3501 et seq.

¶¶¶¶ Among the 1,943 sampled children and adolescents with COVID-19–associated hospitalizations during July 1–December 18, 2021, a total of 1,834 (94.4%) had data available on hospital length of stay, ICU admission, receipt of IMV, and in-hospital death at the time of reporting.

***** Among the 281 sampled children and adolescents with COVID-19–associated hospitalizations during December 19–31, 2021, a total of 266 (94.7%) had data available on hospital length of stay, ICU admission, receipt of IMV, and in-hospital death at the time of reporting.

†††††
https://www.cdc.gov/vaccines/hcp/acip-recs/vacc-specific/covid-19.html

§§§§§
https://covid.cdc.gov/covid-data-tracker/#vaccination-demographics-trends

¶¶¶¶¶
https://www.cdc.gov/coronavirus/2019-ncov/variants/omicron-variant.html

******
https://www.cdc.gov/coronavirus/2019-ncov/vaccines/stay-up-to-date.html
